# Naturally occurring hotspot cancer mutations in Gα_13_ promote oncogenic signaling

**DOI:** 10.1074/jbc.AC120.014698

**Published:** 2021-01-13

**Authors:** Marcin Maziarz, Anthony Federico, Jingyi Zhao, Lorena Dujmusic, Zhiming Zhao, Stefano Monti, Xaralabos Varelas, Mikel Garcia-Marcos

**Affiliations:** 1 Department of Biochemistry, Boston University School of Medicine, Boston, Massachusetts, USA; 2 Section of Computational Biomedicine, Boston University School of Medicine, Boston, Massachusetts, USA

**Keywords:** GTPase, G-protein, G-protein-coupled receptor (GPCR), cancer biology, oncogene, Bladder Cancer

## Abstract

Heterotrimeric G-proteins are signaling switches broadly divided into four families based on the sequence and functional similarity of their Gα subunits: G_s_, G_i/o_, G_q/11_, and G_12/13_. Artificial mutations that activate Gα subunits of each of these families have long been known to induce oncogenic transformation in experimental systems. With the advent of next-generation sequencing, activating hotspot mutations in G_s_, G_i/o_, or G_q/11_ proteins have also been identified in patient tumor samples. In contrast, patient tumor-associated G_12/13_ mutations characterized to date lead to inactivation rather than activation. By using bioinformatic pathway analysis and signaling assays, here we identified cancer-associated hotspot mutations in Arg-200 of Gα_13_ (encoded by *GNA13*) as potent activators of oncogenic signaling. First, we found that components of a G_12/13_-dependent signaling cascade that culminates in activation of the Hippo pathway effectors YAP and TAZ is frequently altered in bladder cancer. Up-regulation of this signaling cascade correlates with increased YAP/TAZ activation transcriptional signatures in this cancer type. Among the G_12/13_ pathway alterations were mutations in Arg-200 of Gα_13_, which we validated to promote YAP/TAZ-dependent (TEAD) and MRTF-A/B-dependent (SRE.L) transcriptional activity. We further showed that this mechanism relies on the same RhoGEF-RhoGTPase cascade components that are up-regulated in bladder cancers. Moreover, Gα_13_ Arg-200 mutants induced oncogenic transformation *in vitro* as determined by focus formation assays. In summary, our findings on Gα_13_ mutants establish that naturally occurring hotspot mutations in Gα subunits of any of the four families of heterotrimeric G-proteins are putative cancer drivers.

Heterotrimeric G-proteins are critical transducers of signaling triggered by a large family of G-protein–coupled receptors (GPCRs). Essentially, GPCRs promote GTP loading on the α-subunits of G-proteins (1, 2), which triggers signaling downstream. Heterotrimeric G-proteins are composed of a nucleotide-binding Gα subunit and an obligatory Gβγ dimer, and they are classified into four families based on the nature of the Gα subunits. These four families are G_s_, G_i/o_, G_q/11_, and G_12/13_, and Gα subunits of each one of them have distinct actions on specific effectors. For example, G_s_ members stimulate adenylyl cyclase activity, whereas G_i/o_ family members tend to inhibit it; G_q/11_ members stimulate phospholipase C enzymes and a subgroup of RhoGEFs; and G_12/13_ members stimulate a different subgroup of RhoGEFs (3, 4). Signaling is terminated upon GTP hydrolysis mediated by the intrinsic GTPase of Gα subunits.

The role of heterotrimeric G-proteins in cancer-related signaling has been documented for decades. Early studies identified cancer-associated mutations in Gα_s_ that disrupted its GTPase activity, rendering the G-protein constitutively active (5). This seminal finding spurred a wave of studies exploring whether analogous mutations introduced artificially in other Gα subunits would also promote their ability to induce oncogenic transformation. It was found that GTPase-deficient mutants of most, if not all, Gα subunits tested led to oncogenic transformation *in vitro*, regardless of the G-protein family they belonged to. For example, Gα_i2_, Gα_o_, and Gα_z_ (G_i/o_ family); Gα_q_ (G_q/11_ family); and Gα_12_ and Gα_13_ (G_12/13_ family), in addition to Gα_s_ (G_s_ family), all promoted oncogenic transformation as assessed by *in vitro* assays using fibroblasts (6, 7, 8, 9, 10, 11, 12, 13). In most cases, transformation *in vitro* correlated well with tumor growth *in vivo* using mouse xenografts. Thus, one theme emerging from these studies was that enhancement of GPCR/G-protein signaling tends to favor oncogenicity.

Despite these initial observations and the identification of some mutations in G-proteins in tumors (5, 14), only with the recent advent of deep-sequencing techniques has it become obvious that dysregulation of the GPCR/G-protein signaling axis in cancer is highly prevalent (15, 16, 17). The mutational landscape of GPCR/G-protein signaling components in cancer supports the theme that G-protein hyperactivation in cancer tends to be pro-oncogenic. There are many examples of GPCRs that are either overexpressed or contain activating mutations (15, 17, 18, 19, 20), and negative regulators of G-protein activity have also been shown to bear loss-of-function mutations (21). As for G-proteins themselves, it is now known that hyperactive G-protein mutants can be very frequent in certain types of cancers. The most striking example is uveal melanoma, in which ∼90% of tumors contain activating mutations in Gα_q_ (encoded by *GNAQ*) or Gα_11_ (*GNA11*) (22, 23). Similarly, activating mutations in Gα_s_ (*GNAS*) can be as frequent as 70% in certain subtypes of pancreatic ductal carcinomas (24, 25), and activating mutations in Gα_i2_ can be as frequent as 24% in epitheliotropic intestinal T-cell lymphomas (26). Thus, for representative members of three of four families of G-proteins (G_q/11_, G_s_, and G_i/o_) the oncogenic activity *in vitro* caused by artificially introduced mutations has found a counterpart in prevalent mutations in cancer. Interestingly, findings so far suggest that the remaining family of G-proteins (G_12/13_) might be an exception to this trend. For example, mutations in Gα_13_ in some types of lymphoma are frequent, but they lead to inactivation rather than activation (27, 28). This suggests that, at least in these lymphomas, Gα_13_ activity is tumor-suppressive. This is the opposite of the oncogene function previously suggested from experiments *in vitro* with a constitutively active artificial Gα_13_ mutation (7, 9).

Activation of Gα_12_ or Gα_13_ proteins leads to activation of RhoA-dependent transcriptional programs, including those mediated by the activation of the Hippo pathway effectors YAP and TAZ. The cascade of events involves the direct activation of a subgroup of RhoGEFs, composed of p115-RhoGEF, PDZ-RhoGEF, and LARG, by active, GTP-loaded Gα subunits of G_12/13_ proteins, which in turn activates RhoA, RhoB, and RhoC GTPases (29). Through mechanisms that involve the remodeling of the actin cytoskeleton, Rho GTPases induce transcriptional responses that include those regulated by YAP/TAZ, which serve as co-factors for the TEA domain–containing transcription factor family (TEADs), and via myocardin-related transcription factors A and B (MRTF-A/B), which serve as co-activators for the transcriptional factor SRF (30, 31, 32, 33, 34, 35, 36). Although the G_12/13_-YAP/TAZ signaling axis has been shown to be oncogenic in some contexts (35, 36, 37), no cancer-associated mutation that activates Gα_12_ or Gα_13_ has been characterized to date. Prompted by the finding that the G_12/13_-YAP/TAZ signaling axis is up-regulated in bladder cancer, here we characterized the effect of hotspot mutations in Gα_13_ identified in this cancer type. We found that mutations in the Arg-200 of Gα_13_, a residue required to hydrolyze GTP, lead to activation of YAP/TAZ-dependent and MRTF-A/B-dependent transcription through a RhoGEF–Rho GTPase cascade and that they promote oncogenic transformation *in vitro*. This implies that naturally occurring hotspot mutations in Gα subunits of any of the four families of heterotrimeric G-proteins are putative cancer drivers.

## Results and discussion

### G_12/13_ pathway up-regulation correlates with increased Yap/TAZ transcriptional activity in bladder cancer

**Figure 1:**
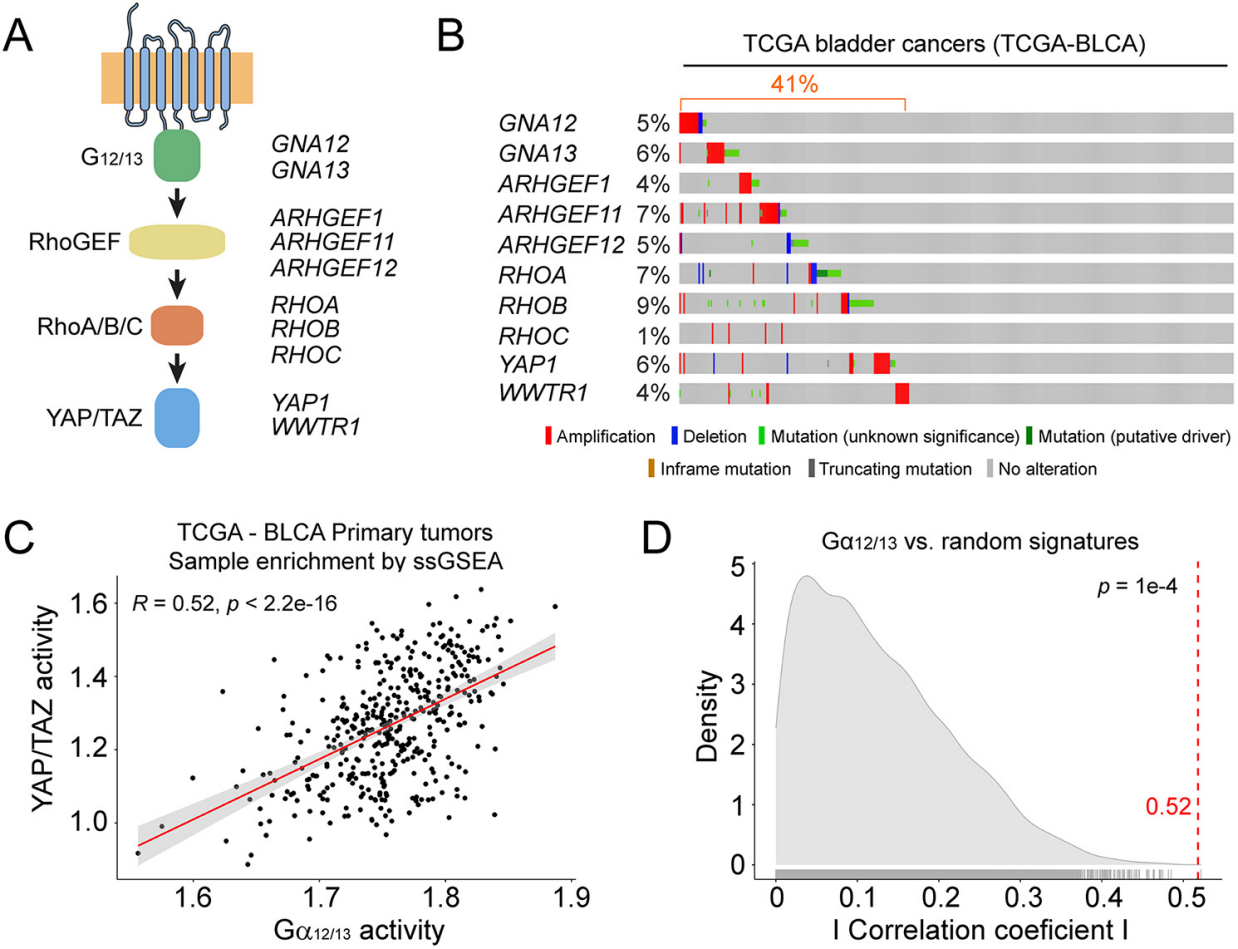
**G_12/13_-YAP/TAZ signaling axis is up-regulated in many bladder cancers.***A*, diagram of a G_12/13_-YAP/TAZ signaling cascade. *B*, many bladder cancers show genetic alterations in the G_12/13_-YAP/TAZ signaling cascade that collectively suggest up-regulation. Data mined from the TCGA-BLCA data set is displayed as an oncoprint representation. *C*, up-regulated expression of the G_12/13_ pathway correlates with YAP/TAZ activation in bladder cancers. The correlation between the expression of G_12/13_ pathway components and a 24-gene transcriptional signature regulated by YAP/TAZ was assessed by ssGSEA. The *solid line* represents the linear regression fit of the data, with 95% confidence intervals indicated in *gray*. *D*, comparison of the correlation coefficient observed in *C* (*vertical dotted line*) with a null distribution generated from bootstrapping correlation coefficients for the G_12/13_ pathway and 10,000 randomly selected 24-gene signatures. The statistical significance *p* value was determined as described under “Experimental procedures.”

We mined data from the Cancer Genome Atlas (TCGA) through cBioportal to explore genomic alterations in components of a G_12/13_-YAP/TAZ pathway (Fig. 1*A*). More specifically, we queried the G-proteins Gα_12_ (*GNA12*) and Gα_13_ (*GNA13*); the RhoGEFs p115-RhoGEF (*ARHGEF1*), PDZ-RhoGEF (*ARHGEF11*), and LARG (*ARHGEF12*); the Rho GTPases RhoA (*RHOA*), RhoB (*RHOB*), and RhoC (*RHOC*); and the Hippo pathway effectors YAP (*YAP1*) and TAZ (*WWTR1*). We found that these genes were altered in a large portion (∼40%) of the TCGA bladder cancers (TCGA-BLCA) (Fig. 1*B*). The alterations appeared to be largely mutually exclusive and trending toward up-regulation. For example, both heterotrimeric G-proteins, two of the three RhoGEFs, and both Hippo effectors displayed amplifications as the dominant feature. For RhoA (*RHOA*) and RhoB (*RHOB*), the main feature was that they were mutated, and several of these mutations are classified as putative drivers in cBioportal (38). Although not all RhoA/RhoB mutations have been characterized, some of them have been previously proposed to lead to signaling activation, like Ala-161 mutations in RhoA (39) or the E172K mutation in RhoB (40). Thus, although LARG (*ARHGEF12*) and RhoC (*RHOC*) are exceptions to the overall trend, these observations suggest that the G_12/13_-YAP/TAZ pathway might be up-regulated in bladder cancer.

Motivated by these observations, we carried out a bioinformatic analysis of gene expression data to establish a potential correlation between up-regulation of the G_12/13_ pathway and YAP/TAZ activation. For this, we turned to a previously characterized 24-gene signature that depends on YAP/TAZ (41) and analyzed its relationship to the expression levels of the rest of the upstream components of the proposed G_12/13_ pathway. We used single-sample gene set enrichment analysis (ssGSEA) to quantify relative enrichment of each pathway across over 400 primary tumors in the TCGA-BLCA RNA-Seq data set. We found a strong correlation between the activation scores of the G_12/13_ pathway and the activation scores for YAP/TAZ (Fig. 1*C*). We then tested the observed correlation coefficient against a null distribution of correlations between ssGSEA-quantified activity of the G_12/13_ pathway and 10,000 random 24-gene signatures, resulting in a significant *p* value of 1e−4 (Fig. 1*D*). Taken together, these observations indicate that up-regulation of the G_12/13_ pathway in bladder cancer correlates with increased transcriptional output of the downstream effectors YAP/TAZ.

### Gα13 Arg-200 mutants induce YAP/TAZ activity via a RhoGEF–Rho GTPase axis

**Figure 2:**
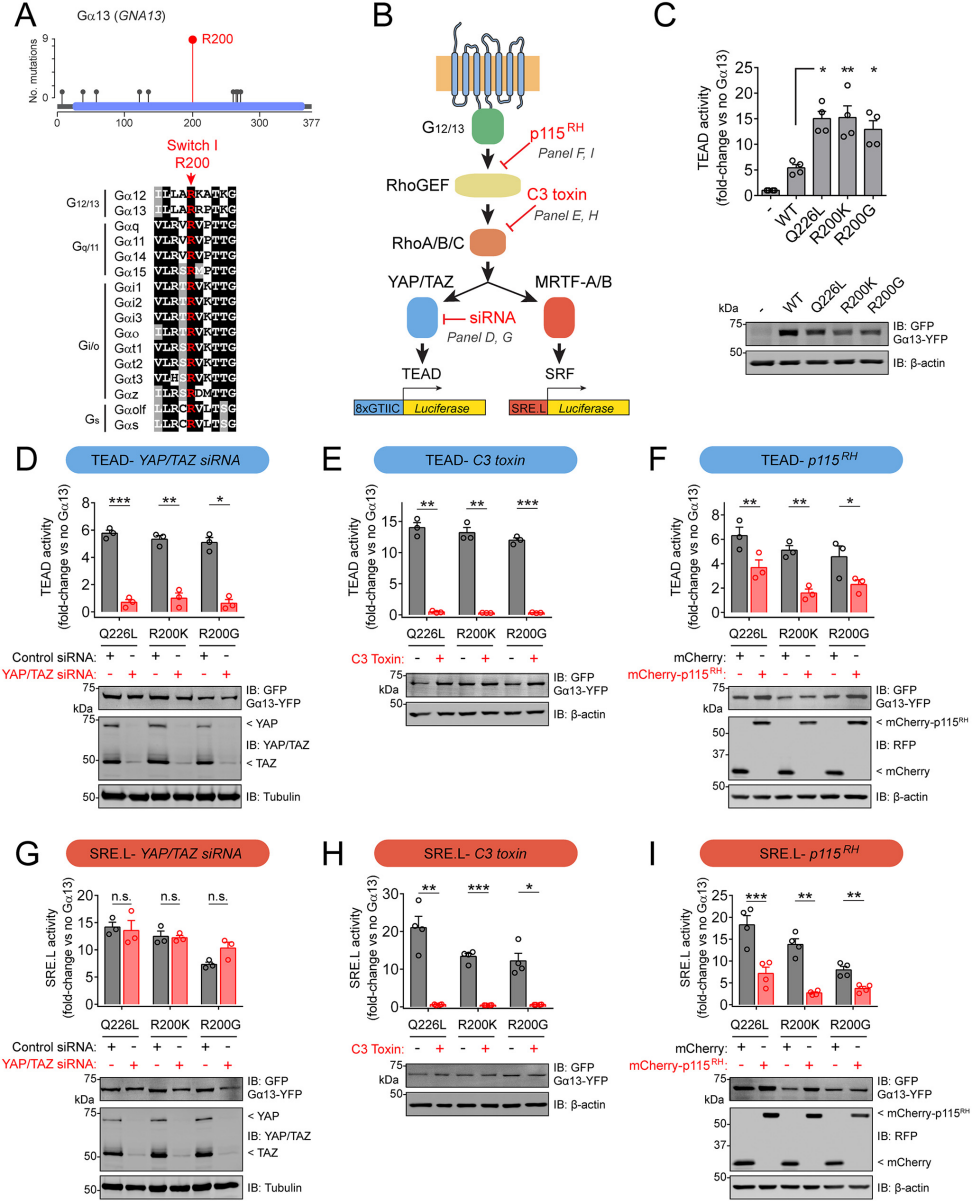
**Hotspot mutations in Gα_13_ Arg-200 cause constitutive G-protein activation and lead to enhanced YAP/TAZ-dependent and MRTF-A/B-dependent transcription.***A*, *top*, lollipop plot of Gα_13_ residues mutated in bladder cancer. *Bottom*, alignment of Gα switch I region showing in *red* the fully conserved arginine that corresponds to Gα_13_ Arg-200. *B*, diagram of a G_12/13_ signaling cascade culminating in the activation of transcriptional regulators and specific luciferase-based reporters used to measure their activity. Manipulations implemented in other *panels* of this figure to inhibit specific steps of the pathway are indicated in *red*. *C*, Gα_13_ Arg-200 mutants activate YAP/TAZ-dependent transcription. HEK293T cells were transfected with plasmids for the expression of the indicated Gα_13_ constructs and TEAD reporter (*8xGTIIC*) activity determined as described under “Experimental procedures.” Results are mean ± S. E. (*error bars*), *n* = 4. *, *p* < 0.05; **, *p* < 0.01, analysis of variance with Tukey post hoc test. *D* and *G*, YAP/TAZ depletion abolishes TEAD reporter (*D*) but not SRE.L reporter (*G*) activation caused by Gα_13_ Arg-200 mutants. HEK293T cells were transfected with plasmids for the expression of the indicated Gα_13_ constructs and with the indicated siRNAs, and TEAD reporter or SRE.L reporter activity was determined as described under “Experimental procedures.” Results are mean ± S. E., *n* = 3. *, *p* < 0.05; **, *p* < 0.01; ***, *p* < 0.001, *n.s.*, not significant, Student's *t* test. *E* and *H*, Rho GTPase blockade abolishes TEAD reporter (*E*) and SRE.L reporter (*H*) activation caused by Gα_13_ Arg-200 mutants. HEK293T cells were transfected with plasmids for the expression of the indicated Gα_13_ constructs with or without a plasmid for the expression of C3 toxin. TEAD reporter or SRE.L reporter activity was determined as described under “Experimental procedures.” Results are mean ± S. E., *n* = 3–4. *, *p* < 0.05; **, *p* < 0.01; ***, *p* < 0.001, Student's *t* test. *F* and *I*, blocking Gα_13_-mediated activation of RhoGEFs with a dominant-negative construct (p115^RH^) inhibits TEAD reporter (*F*) and SRE.L reporter (*I*) activation caused by Gα_13_ Arg-200 mutants. HEK293T cells were transfected with plasmids for the expression of the indicated Gα_13_ constructs and a plasmid for the expression of mCherry-p115^RH^ or mCherry as negative control. TEAD reporter or SRE.L reporter activity was determined as described under “Experimental procedures.” Results are mean ± S. E., *n* = 3–4. *, *p* < 0.05; **, *p* < 0.01; ***, *p* < 0.001, Student's *t* test. For all *panels* showing reporter activation results, an immunoblot of lysates of cells used in one of the experiments is shown *below* the graph.

Although overexpression of WT G_12/13_ family Gα proteins has been found before to be sufficient to promote transformation (7, 8), a recent study also found that the mutation frequency of *GNA13* in the TCGA-BLCA data set is statistically higher than background mutation frequency (*q* = 0.007) (42). Moreover, the distribution of mutations across the sequence of Gα_13_ suggested a hotspot at Arg-200 (Fig. 2*A*). The presence of an arginine in this position is absolutely conserved across Gα subunits (Fig. 2*A*), and its mutation in several other Gα subunits leads to increased activity and favors oncogenic transformation (5, 11, 13, 14). From studies in other Gα proteins, it has been found that this arginine is crucial for GTPase activity and that it cannot be replaced by other amino acids, even if they preserve the positive charge of the side chain like in the case of lysine (5, 43, 44, 45). Thus, we hypothesized that Gα13 Arg-200 mutations identified in bladder cancer would similarly induce the formation of an active G-protein that increases downstream signaling, including YAP/TAZ (Fig. 2*B*). Because mutation of this arginine to any other residue is expected to have similar consequences (5, 43), we focused our efforts on characterizing Gα_13_ R200K and Gα_13_ R200G because these are the two mutants most frequently found in bladder cancer. Before assessing the impact of these mutants in cell signaling assays, we validated that they adopted an active conformation by using a well-validated assay that relies on protection from trypsin hydrolysis (Fig. S1) (44, 46). Next, we expressed Gα_13_ R200K and Gα_13_ R200G in HEK293T cells and assessed activation of YAP/TAZ using a TEAD reporter assay (Fig. 2*C*). We compared the effect of expressing these two mutants with that of Gα13 WT as well as with that of Gα13 Q226L, an artificial mutant previously shown to enhance downstream signaling including YAP/TAZ-dependent TEAD transcriptional activity (34, 37). Whereas expression of Gα_13_ WT led to a modest increase of TEAD activity, expression of Gα_13_ R200K and Gα_13_ R200G led to a significantly larger increase comparable with that observed in cells expressing the control mutant Gα_13_ Q226L (Fig. 2*C*). To determine whether the observed increase in TEAD activity by Gα_13_ mutants was mediated by YAP/TAZ, we knocked down both proteins simultaneously using a previously validated siRNA sequence (47, 48). As expected, depletion of YAP and TAZ led to a large suppression of TEAD activation by Gα_13_ R200K, R200G, or Q226L (Fig. 2*D*). To further map the cascade of events leading to YAP/TAZ activation by Gα_13_ mutants, we blocked the pathway that putatively operates in bladder cancer at different levels. First, inhibition of the Rho GTPases RhoA, RhoB, and RhoC by expression of *Clostridium botulinum* C3 toxin efficiently suppressed TEAD activation by Gα_13_ R200K, R200G, or Q226L (Fig. 2*E*). Then we tested the effect of a fragment of p115-RhoGEF that works as a dominant-negative by preventing the binding of active Gα_13_ to its target RhoGEFs that operate upstream of Rho GTPases in the pathway (49). Expression of this dominant-negative construct, consisting of p115-RhoGEF's RGS homology (RH) domain (p115^RH^), but not a control construct, inhibited TEAD activation by Gα_13_ R200K, R200G, or Q226L (Fig. 2*F*). To further validate the specificity of these manipulations, we tested their impact on Gα_13_-mediated activation of another transcriptional output not controlled by YAP/TAZ but still dependent on Rho GTPase activation (*i.e.* the transcriptional activation of SRF via MRTF-A/B) (Fig. 2*B*). As expected, bladder cancer–associated mutants Gα_13_ R200K and R200G led to robust activation of the SRF reporter, comparable with that of the positive control Gα_13_ Q226L, which was suppressed by inhibition of the activation of RhoGEFs or Rho GTPases but not upon YAP/TAZ depletion (Fig. 2, *G–I*). Taken together, these results demonstrate that Gα_13_ hotspot mutations in Arg-200 found in bladder cancer are *bona fide* activating mutations that lead to induction of YAP/TAZ-dependent transcription via a RhoGEF–Rho GTPase cascade.

### Gα_13_ Arg-200 mutants induce oncogenic transformation in vitro

**Figure 3:**
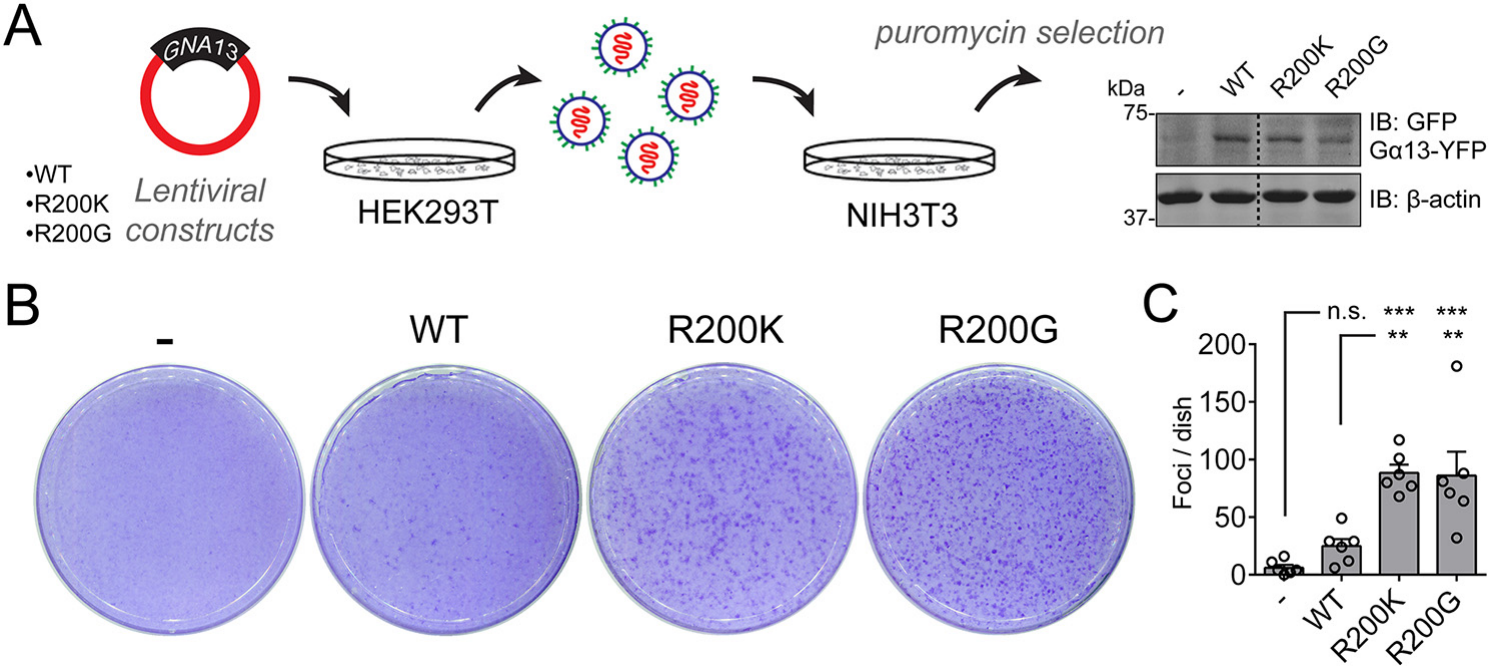
**Gα_13_ Arg-200 mutants induce NIH3T3 cell transformation *in vitro*.***A*, generation of NIH3T3 cell lines stably expressing the indicated Gα_13_ proteins. Lentiviral particles for the expression of Gα_13_ were generated in HEK293T cells and used to transduce NIH3T3 cells, followed by antibiotic selection. Lysates of each one of the cell lines were immunoblotted as indicated. Images were generated by splicing lanes from the same membrane, and the *vertical dotted line* indicates the position of the boundary between the two segments that were merged. *B* and *C*, Gα_13_ Arg-200 mutants promote focus formation in NIH3T3 cells more efficiently than Gα_13_ WT. Cells were seeded on plates and stained with crystal violet 10 days later. Images of a representative experiment are shown in *B*, whereas *C* shows the quantification of foci. Results are mean ± S. E. (*error bars*), *n* = 6. **, *p* < 0.01; ***, *p* < 0.001; *n.s.*, not significant, analysis of variance with Tukey post hoc test.

Finally, we sought to determine whether the Gα_13_ hotspot mutations in Arg-200 described above would be sufficient to promote oncogenic transformation *in vitro*. For this, we used focus formation assays with NIH3T3 cells. This widely used system is particularly well-suited to analyze the putative oncogenic activity of Gα_13_ Arg-200 mutants because it has been used for the vast majority of Gα oncogenic mutants reported to date as a good proxy for tumor growth in mice, including for the oncogenic activity of artificial activating mutations introduced in Gα_13_ (7). First, we assessed whether Gα_13_ R200K and Gα_13_ R200G mutants also lead to increased signaling activity in NIH3T3 cells. Surprisingly, we found that whereas Gα_13_ R200K and Gα_13_ R200G led to robust increases in the MRTF-A/B-dependent SRE.L reporter, they had no significant effect on the activity of the YAP-TAZ–dependent TEAD reporter (Fig. S2). These results confirm that Gα_13_ R200K and Gα_13_ R200G behave as active G-proteins but that the downstream signaling consequences are cell type–specific. Next, we generated NIH3T3 cell lines stably expressing Gα_13_ WT, Gα_13_ R200K, and Gα_13_ R200G at comparable levels by lentiviral transduction and selection with the appropriate agents (Fig. 3*A*). Both Gα_13_ R200K and Gα_13_ R200G induced the formation of numerous foci, whereas Gα_13_ WT only had a modest effect (Fig. 3, *B* and *C*).

### Conclusions

Recent reports have suggested that mutations in Gα_13_ are putative oncogene drivers in bladder cancer based on bioinformatics predictions (17, 42, 50), but no other experimental evidence to support the predictions has been provided. The results presented here provide the missing experimental evidence that supports the idea of Gα_13_ hotspot mutations as putative drivers in bladder cancer and suggest that pharmacological blockade of the pathway activated downstream might be a viable therapeutic avenue. Moreover, our findings on Gα_13_ mutants establish that naturally occurring hotspot mutations in Gα subunits of any of the four families of heterotrimeric G-proteins (*i.e.* in G_s_, G_i/o_, G_q/11,_ and, now, G_12/13_) are putative cancer drivers, thereby providing definitive confirmation of a long-held tenet.

## Experimental procedures

### Data processing

Data for the oncoprint in Fig. 1*B* were obtained through cBioportal (38) by querying the term *GNA13* on March 23rd, 2020 in the data set “Bladder Cancer (TCGA, Cell 2017).” For the lollipop plot in Fig. 2*A*, data were obtained from all of the data sets classified as Bladder Urothelial Carcinoma in cBioportal. TCGA-BLCA RNA-Seq count matrix (generated with STAR 2-Pass and HTSeq-Counts) and available metadata were downloaded through the Genomic Data Commons gdc-client (51, 52). We performed a variance-stabilizing transformation of the data using the R package DESeq2 (version 1.23.10) followed by a log transformation (53).

### Pathway-level correlation analysis

Pathway activity in TCGA-BLCA was measured for G_12/13_ and YAP/TAZ signatures—represented by key selected genes (for G_12/13_: *GNA12, GNA13, ARHGEF1, ARHGEF11, ARHGEF12, RHOA, RHOB,* and *RHOC*; for YAP/TAZ: *YAP1, WWTR1, MYOF, AMOTL2, LATS2, CTGF, CYR61, ANKRD1, ASAP1, AXL, F3, IGFBP3, CRIM1, FJX1, FOXF2, GADD45A, CCDC80, NT5E, DOCK5, PTPN14, ARHGEF17, NUAK2, TGFB2*, and *RBMS3*)—through gene set variation analysis using the ssGSEA method and a Gaussian kernel from the R package GSVA (version 1.34.0) (54). We then measured the Pearson correlation between the activities of each pathway across all primary tumors. We further tested the significance of the observed correlation coefficient by comparing it with a null distribution generated through bootstrapping 10,000 random 24-gene signatures and measuring the correlation of their ssGSEA-quantified activity with the G_12/13_ pathway.

### Plasmid constructs and siRNAs

pGL3b-8xGTIIC-luciferase (TEAD reporter) (55) was from Addgene (catalog no. 34615). pGL3-SRE.L (56) was a gift from Richard Neubig (Michigan State University). pCMV-Beta (Clontech, 631719) was a gift from Matthew Layne (Boston University). Plasmid pCS2-Nanoluc encoding nanoluciferase driven by the cytomegalovirus promoter was a gift from Daniel Cifuentes (Boston University). pcDNA3.1-Gα_13_(EE) was from the cDNA Resource Center (GNA130EI00). pcDNA3.1-Gα_13_(EE)-YFP was generated as described previously (57). Lentiviral pLVX-puro-Gα_13_(EE)-YFP plasmids were generated by inserting Gα_13_(EE)-YFP into the EcoRI site of a modified pLVX-puro plasmid (pLVX-puro-MCS+). Plasmid pEF-C3 was a gift from Silvio Gutkind (University of California, San Diego). mCherry-p115^RH^ (also known as Lck-mCherry-RGS (49)) and mCherry (also known as Lck-mCherry) plasmids were a gift from Joachim Goedhart (University of Amsterdam). All point mutations were generated using QuikChange II (Agilent, 200523). siRNA used for knockdown of YAP and TAZ was UGUGGAUGAGAUGGAUACA (47, 48), and the control siRNA was from Qiagen (catalog no. 1027310).

### Trypsin protection assays

This assay was carried out as described previously (29), except that HEK293T lysates were treated with trypsin for 5 min.

### Reporter assays in HEK293T and NIH3T3 cells

TEAD and SRE.L reporter assays in HEK293T cells (ATCC, CRL-3216) were performed as described previously (58) using the Dual-Glo Luciferase Assay System (Promega, E2920) to determine luciferase activity and fluorescein di-β-d-galactopyranoside (Marker Gene Technologies) for β-gal activity. Cells were transfected with the following plasmids using calcium phosphate: pGL3-SRE.L (0.5 μg) or pGL3b-8xGTIIC-luciferase (0.5 μg) and pCMV-Beta (0.5 μg), along with plasmid pcDNA3.1-Gα_13_(EE)-YFP (0.6 μg of WT or 0.15–0.2 μg of mutant plasmid per well). In some experiments, 0.1 μg of pEF-C3, mCherry-p115^RH^, or mCherry was also co-transfected. Six hours after transfection, medium was replaced by Dulbecco's modified Eagle's medium with 0.5% fetal bovine serum. 16-24 h later, cells were washed with PBS and harvested by gentle scraping. When using siRNA, cells were first reverse-transfected with 20 pmol of YAP/TAZ or control siRNA using Lipofectamine RNAiMAX (Invitrogen, 13778075) the day before calcium phosphate transfection. For NIH3T3 cells (ATCC, CRL-1658), the same procedures were followed except that the plasmids pGL3-SRE.L (0.25 μg) or pGL3b-8xGTIIC-luciferase (0.25 μg), pCS2-Nanoluc (0.05 μg), and 0.5 μg of WT or 0.3–0.6 μg of mutant plasmid per well were transfected using Turbofect (ThermoScientific, R0531) and that firefly luciferase and nanoluciferase activities were determined using the Nano-Glo Dual-Luciferase reporter assay system (Promega, N1610).

### NIH3T3 focus formation

Lentivirus packaging, transduction, and selection (1 μg/ml puromycin) were carried out as described previously (57). All surviving clones were pooled and maintained in the presence of 0.5 μg/ml puromycin. For focus formation assays, NIH3T3 cell lines were seeded (200,000 cells/plate) in 10-cm plates coated with 0.1% gelatin. Medium was replaced at days 5, 7, and 9 after seeding. At day 10, cells were washed with cold PBS, fixed with cold 100% methanol, and stained with crystal violet (0.05% (w/v) in 20% (v/v) methanol). After washing with 20% (v/v) methanol, plates were imaged using a flatbed scanner, and distinct visible colonies (∼1.5 mm^2^ or larger) with dark blue staining were manually counted in the whole plate.

### Immunoblotting

Cell pellets were lysed and immunoblotted as described previously (58) using the following antibodies: GFP (1:1,000; Clontech JL-8), red fluorescent protein (1:1,000; Rockland Immunochemicals 600-401-379), YAP/TAZ (1:1,000; Cell Signaling Technology, D24E4), α-tubulin (1:2,500; Sigma T6074), β-actin (1:2,000; LI-COR 926-42212), and Glu-Glu (1:1,000; Biolegend 901801). The secondary antibodies were goat anti-rabbit Alexa Fluor 680 (1:10,000; Life Technologies A21077), goat anti-mouse Alexa Fluor 680 (1:10,000; Life Technologies A28183), goat anti-mouse IRDye 800 (1:10,000; LI-COR 926-32210), and goat anti-rabbit IRDye 800 (1:10,000; LI-COR 926-32211).

## Data availability

All data are contained in the article except the raw data used for the genomics analysis, which corresponds to the Cancer Genome Atlas data set named TCGA-BLCA and was accessed and/or downloaded directly from cBioPortal or Genomic Data Commons as indicated under “Experimental procedures.”

10.13039/100000057HHS | NIH | National Institute of General Medical Sciences (NIGMS) (R01GM130120) to Mikel Garcia-Marcos
10.13039/100000048American Cancer Society (ACS) (PF-19-084-01-CDD) to Marcin Maziarz
10.13039/100000048American Cancer Society (ACS) (RSG-17-138-01-CSM) to Xaralabos Varelas
10.13039/100000050HHS | NIH | National Heart, Lung, and Blood Institute (NHLBI) (R01HL12439) to Xaralabos Varelas
