# Supplementary material for: Naturally occurring hotspot cancer mutations in Gα13 promote oncogenic signaling
Source: J Biol Chem. 2021 Jan 13;295(49):16897–904. doi: 10.1074/jbc.AC120.014698 (PMC7864081; doi:10.1074/jbc.AC120.014698)
Supplement: Supplementary file 1 [file mmc1.docx]

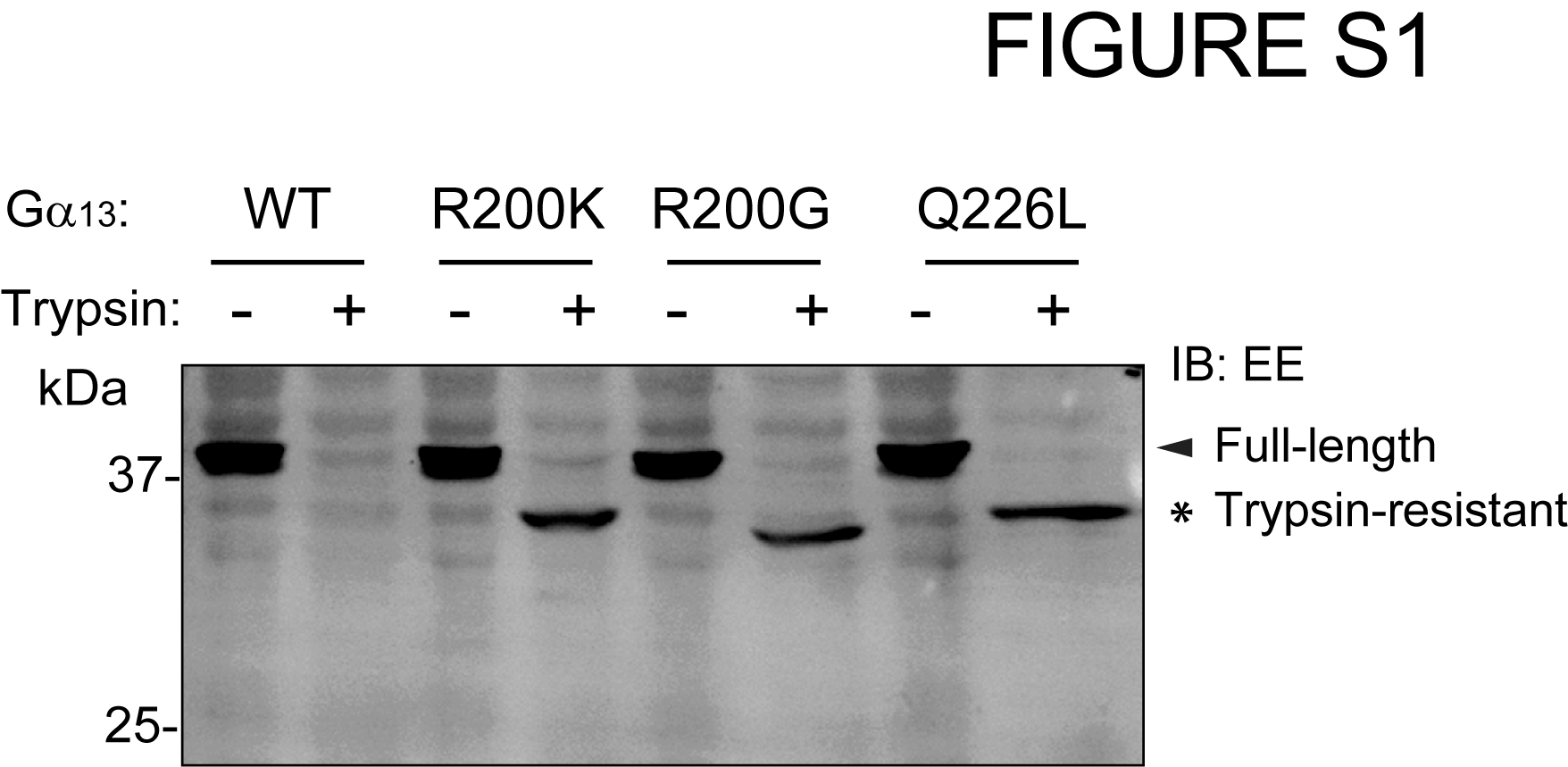


**Figure S1. Gα_13_ R200K and Gα_13_ R200G mutants adopt an active conformation as determined by protection from trypsin-mediated hydrolysis.** Lysates of HEK293T cells expressing the indicated Gα_13_ mutants were incubated with (+) or without (-) trypsin and analyzed by immunoblotting. Arrowhead indicates full-length Gα_13_, and “*” indicates a trypsin-resistant fragment of active Gα_13_ generated after cleavage of a small N-terminal fragment. Whereas Gα_13_ WT, presumably GDP-bound, is readily digested by trypsin, Gα_13_ R200K and Gα_13_ R200G yield an N-terminally cleaved fragment like that observed in the GTPase-deficient Gα_13_ Q226L mutant. Results representative of two independent experiments are presented.


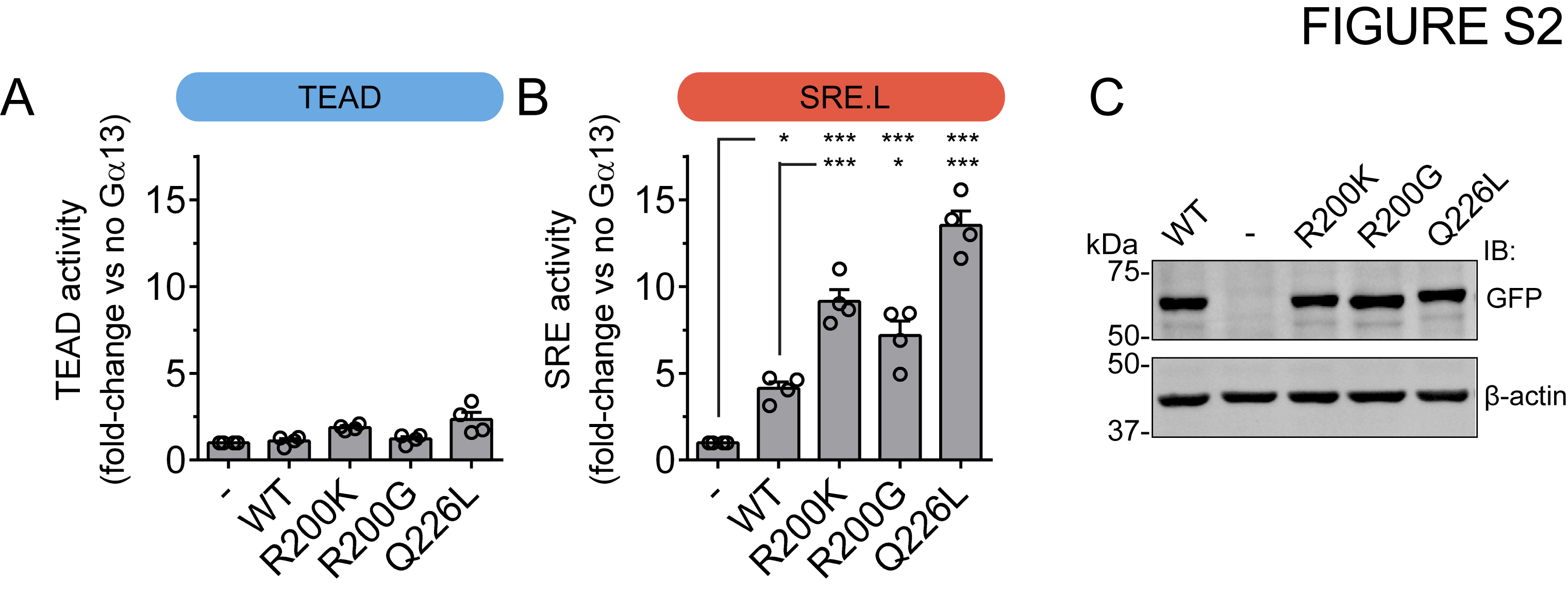


**Figure S2. Hotspot mutations in Gα_13_ Arg-200 lead to enhanced SRE.L but not TEAD transcriptional reporter activity in NIH3T3 cells.**  NIH3T3 cells were transfected with plasmids for the expression of the indicated Gα_13_ constructs, and TEAD reporter (A) or SRE.L reporter (B) activity was determined as described in “*Experimental procedures*”. Mean ± S.E.M, n= 4. *p<0.05, ***p<0.001, ANOVA with Tukey post hoc test. All Gα_13_ constructs were expressed at similar levels as determined by immunoblotting (C).
